# Is the postoperative pedicle screw position after dorsal instrumentation with or without intraoperative cone beam CT imaging worse in patients with obesity than in normal-weight patients?

**DOI:** 10.1186/s13018-022-03369-x

**Published:** 2022-11-03

**Authors:** Felix Zimmermann, Katharina Kohl, Eric Mandelka, Paul A. Grützner, Jochen Franke, Sven Y. Vetter

**Affiliations:** BG Klinik Ludwigshafen, Ludwig-Guttmann-Straße 13, 67071 Ludwigshafen am Rhein, Germany

**Keywords:** Spine, Dorsal instrumentation, Pedicle screw, Cone-beam CT, Patients with obesity, BMI

## Abstract

**Background:**

Intraoperative cone beam CT (CBCT) imaging in dorsal instrumentation facilitates pedicle screw positioning. However, in patients with obesity, the benefit may be reduced due to artifacts that affect image quality. The purpose of this study was to evaluate whether intraoperative CBCT leads to an improved postoperative screw position compared to conventional fluoroscopy independent of body weight.

**Methods:**

A total of 71 patients (18 patients with a BMI > 30 kg/m^2^, 53 patients with a BMI < 30 kg/m^2^) who underwent dorsal instrumentation with intraoperative CBCT imaging were included in study groups one (SG1) and two (SG2). Two control groups (CG1 and CG2) were randomly sampled to include 22 patients with a BMI > 30 kg/m^2^ and 60 patients with a BMI < 30 kg/m^2^ who underwent dorsal instrumentation without intraoperative CBCT imaging. The pedicle screw position in postoperative computed tomography was assessed using the Gertzbein–Robbins classification.

**Results:**

In SG1 (BMI > 30 kg/m^2^), a total of 107 (83.6%) pedicle screws showed no relevant perforation (type A + B), and 21 (16.4%) pedicle screws showed relevant perforation (type C − E). In SG2 (BMI < 30 kg/m^2^), 328 (90.9%) screws were classified as type A + B, and 33 (9.1%) screws were classified as type C − E. In CG1 (BMI > 30 kg/m^2^), 102 (76.1%) pedicle screws showed no relevant perforation (type A + B), and 32 (23.9%) pedicle screws showed relevant perforation (type C − E). In CG2 (BMI < 30 kg/m^2^), 279 (76.9%) screws were classified as type A + B, and 84 (23.1%) screws were classified as type C − E. There were significant differences between the values of SG1 and SG2 (*p* = 0.03) and between the values of SG2 and CG2 (*p* < 0.0001).

**Conclusion:**

CBCT imaging in dorsal instrumentation can lead to an improved pedicle screw position among both patients with obesity and normal-weight patients. However, patients with obesity showed significantly worse pedicle screw positions postoperatively after dorsal instrumentation with intraoperative CBCT imaging than normal-weight patients.

## Background

Pedicle screw placement in dorsal instrumentation without perforating the pedicle can be challenging. The percentage frequency of pedicle screw perforation in the currently available literature varies between 1.5 and 55% [1–4]. One reason seems to be that intraoperative assessment of the pedicle screw position with conventional fluoroscopy is difficult, which often leads to a particular degree of uncertainty in the assessment. Since the intraoperative pedicle screw position can be assessed much more clearly in computed tomography (CT), it is increasingly recommended to use cone beam computed tomography (CBCT) or computed tomography intraoperatively (iCT) [5–8]. Moreover, it has been shown that intraoperative imaging with CBCT or iCT during dorsal instrumentation can lead to improved pedicle screw placement and low postoperative revision rates [9–11]. However, an especially challenging situation arises with the dorsal instrumentation of patients with obesity. The WHO defines obesity as a body mass index (BMI) greater than 30 kg/m^2^ [12]. Various factors, such as the need for longer surgical instruments and more difficult surgical approaches, contribute to aggravated conditions among patients with obesity [13]. Furthermore, among patients with obesity, the image quality of intraoperative imaging is additionally reduced by enhanced artifact formations triggered by increased density of adipose tissue [14]. Consequently, it is more difficult to assess images, which may be due to increased postoperative screw malposition.

Therefore, the purpose of this retrospective study was to evaluate whether the use of intraoperative CBCT imaging leads to an improved postoperative pedicle screw position compared to conventional fluoroscopy independent of body weight in patients with obesity and normal-weight patients. The hypotheses were as follows: (1) The use of intraoperative CBCT imaging in dorsal instrumentation in patients with obesity and normal-weight patients may reduce the rate of misplaced screws on postoperative CT, and (2) patients with obesity show worse postoperative pedicle screw positions.

## Methods

This retrospective study obtained approval from the local ethics committee (Reference no. 2020-15452-retrospektiv). A total of 18 patients with a BMI > 30 kg/m^2^ (age 64.1 ± 19.6 [27–92] years; m/f (10/8); BMI 34.8 ± 4.0 kg/m^2^) who underwent dorsal instrumentation with intraoperative CBCT imaging receiving 128 pedicle screws in a trauma center level one between March 2015 and December 2018 were included in study group one (SG1) of this retrospective analysis. Simultaneously, a total of 53 patients with BMI < 30 kg/m^2^ (age 63.0 ± 19.7 [22–98] years; m/f (36/17); BMI 25.5 ± 3.0 kg/m^2^) who underwent dorsal instrumentation with intraoperative CBCT imaging receiving 361 pedicle screws were included in study group two (SG2).

Based on the sample size of SG1, control group one (CG1) was established with 22 randomly selected patients with a BMI > 30 kg/m^2^ (age 67.2 ± 10.7 [38–85] years; m/f (14/8); BMI 33.3 ± 2.6 kg/m^2^) who received 134 pedicle screws without intraoperative CBCT imaging during the same period from January 2017 to January 2019. In addition, control group two (CG2) was established with 60 patients with BMI < 30 kg/m^2^ (age 65.3 ± 15.5 [24–92] years; m/f (37/23); BMI 24.8 ± 2.7 kg/m^2^) who received 363 screws without intraoperative CBCT imaging during the same time period as the patients in CG1.

The SG groups were formed from all patients who underwent operative treatment using intraoperative CBCT imaging during the reported time period. The CG groups were formed age- and sex-adapted to the SG groups from the available data of the clinic internal database.

In all patients, pedicle screws were inserted and cannulated over previously inserted Kirschner wires during dorsal instrumentation, and the intraoperative imaging procedure was performed as previously described [15]. The decision whether intraoperative CBCT imaging was performed was made by the surgeon. For intraoperative CBCT imaging in SG patients, two different models were used: Cios Spin (Siemens, Erlangen, Germany) and ARCADIS Orbic 3D (Siemens, Erlangen, Germany). Pedicle screw position was assessed intraoperatively using the Gertzbein–Robbins classification [16] modified from Schatlo et al. [17]. Pedicle screws with a deviation of more than 2 mm (Gertzbein–Robbins type C − E) according to Schatlo et al. [17] outside the pedicle were classified as perforated and in need of intraoperative revision. If malpositioned pedicle screws were corrected, intraoperative fluoroscopy and another intraoperative CBCT scan were performed afterward. This procedure was continued until a satisfactory result was determined. Postoperative CT (Aquilion Prime SP and Aquilion Lightning, Canon, Tokyo, Japan) was performed in all patients to verify the surgical outcome.

In this study, we retrospectively evaluated the pedicle screw position in the postoperative CT of SG1, SG2, CG1 and CG2 and of the subgroups of SG1 and CG1. The pedicle screw position was assessed postoperatively using the Gertzbein–Robbins classification [16] modified from Schatlo et al. [17]. Gertzbein–Robbins types A + B were classified as not perforating the pedicle, and types C − E were categorized as perforating the pedicle.

Patient BMI was defined according to the WHO recommendations [12], and SG1 and CG1 were divided into two subgroups each: BMI 30–35 kg/m^2^ and BMI > 35 kg/m^2^.

The data are presented as the means ± standard deviations (ranges) and as absolute values (relative values). Cochran–Mantel–Haenszel and contingency tables were used to assess the differences between the study group, the control group and their subgroups. All analyses were performed using GraphPad Prism (version 9.3.1; GraphPad Software, San Diego, CA, USA) and Minitab® (version 21.2.; Minitab Inc., Pennsylvania, USA). The significance level was set at 0.05.

## Results

The postoperative CT of SG1 (BMI > 30 kg/m^2^) showed 107 (83.6%) pedicle screws without relevant perforation (type A + B) and 21 (16.4%) pedicle screws with a relevant perforation (type C − E). In SG2 (BMI < 30 kg/m^2^), 328 (90.9%) screws were postoperatively classified as type A + B, and 33 (9.1%) screws were classified as type C − E. There was a significant difference between the values of SG1 and SG2 (*p* = 0.04).

In CG1 (BMI > 30 kg/m^2^), 102 (76.1%) pedicle screws showed no relevant perforation (type A + B), and 32 (23.9%) pedicle screws showed relevant perforation (type C − E) on postoperative CT. In CG 2 (BMI < 30 kg/m^2^), 279 (76.9%) pedicle screws were postoperatively classified as type A + B, and 84 (23.1%) screws were classified as type C − E. There was also a significant difference between the values of SG2 and CG2 (*p* < 0.0001), but there were no statistically significant differences between the other values.

Most of the type C − E pedicle screws in SG1, SG2, CG1 and CG2 showed lateral deviation of the pedicle (Table 1).

**Table 1:** Number of medial and lateral deviation type C − E pedicle screws in SG1, SG2, CG1 and CG2

	Medial deviation	Lateral deviation
SG1 (*n* = 21)	6 (28.6%)	15 (71.4%)
SG2 (*n* = 26)	14 (42.4%)	19 (57.6%)
CG1 (*n* = 32)	13 (40.6%)	19 (59.4%)
CG2 (*n* = 83)	32 (38.1%)	52 (61.9%)

The absolute and relative frequencies of medial and lateral deviations of type C − E pedicle screws

The demographics and the pedicle screw position in the SG1 and CG1 subgroups are shown in Table 2. The CG1 patients with a BMI > 35 kg/m^2^ who received pedicle screws with conventional fluoroscopy showed the highest number of type C − E screw perforations. There was no significant difference between the subgroups.

**Table 2:** Demographics and pedicle screw position in the SG1 and CG1 subgroups

	Number of patients	Age	Male/Female	Number of pedicle screws	Type A + B	Type C − E
Subgroup 1 of SG1 (BMI 30–35 kg/m^2^)	10	65.1 ± 23.7 [27–92]	6/4	68	57 (83.8%)	11 (16.2%)
Subgroup 2 of SG1 (BMI > 35 kg/m^2^)	8	63.0 ± 13.5 [42–84]	4/4	60	50 (83.3%)	10 (16.7%)
Subgroup 1 of CG1 (BMI 30–35 kg/m^2^)	17	65.8 ± 10.8 [38–81]	11/6	92	72 (78.3%)	20 (21.7%)
Subgroup 2 of CG1 (BMI > 35 kg/m^2^)	5	70.2 ± 10.1 [65–85]	3/2	42	30 (71.4%)	12 (28.6%)

The mean values ± standard deviations [range] and absolute and relative frequencies are shown. Pedicle screw position was assessed using the Gertzbein–Robbins classification [16] modified from Schatlo et al. [17]

The majority of pedicle screws in all main groups (SG1, SG2, CG1, CG2) were inserted into the thoracic segments of the spine. While 14.1% and 38.5% of pedicle screws were also inserted into the cervical spine in SG1 and SG2, respectively, no pedicle screws in CG1 and only 8.3% of pedicle screws in CG2 were inserted into the cervical spine (Table 3).

**Table 3:** Absolute and relative distribution of pedicle screws among spinal segments in SG1, SG2, CG1 and CG2

	Cervical segment	Thoracal segment	Lumbar segment
SG1 (*n* = 128)	18 (14.1%)	70 (54.7%)	40 (31.3%)
SG2 (*n* = 361)	139 (38.5%)	170 (47.1%)	52 (14.4%)
CG1 (*n* = 134)	–	70 (52.2%)	64 (47.8%)
CG2 (*n* = 363)	30 (8.3%)	177 (48.8%)	156 (43.0%)

Absolute and relative frequencies

## Discussion

The purpose of this retrospective study was to evaluate whether the use of intraoperative imaging by cone beam CT (CBCT) leads to an improved postoperative pedicle screw position compared to conventional fluoroscopy independent of body weight in patients with obesity and normal-weight patients. The hypotheses were that (1) the use of intraoperative CBCT imaging in dorsal instrumentation in patients with obesity and normal-weight patients may reduce the rate of misplaced screws on postoperative CT and that (2) patients with obesity show worse postoperative pedicle screw positions.

The results of this retrospective study suggest that (1) intraoperative CBCT imaging in comparison with conventional fluoroscopy leads to an improved postoperative screw position among both patients with obesity and normal-weight patients. However, (2) the patients with obesity showed significantly worse pedicle screw positions postoperatively after dorsal instrumentation with intraoperative CBCT imaging than normal-weight patients.

Moreover, in the analysis of subgroups, the patients with obesity, especially those with BMI > 35 kg/m^2^, who received pedicle screws with intraoperative conventional fluoroscopy had postoperatively higher rates of pedicle screw perforations compared to those patients who received intraoperative CBCT imaging. However, the differences between the subgroups of the patients with obesity were not statistically significant. Furthermore, the results suggest that the normal-weight patients who received pedicle screws with intraoperative CBCT imaging showed significantly better pedicle screw positions postoperatively than those patients who received only conventional fluoroscopy intraoperatively. However, even despite the intraoperative CBCT scans, perforation rates of 16.4% and 8.7% were still observed in the SG1 and SG2 groups. Simultaneously, it was also shown that the perforations were mainly lateral perforations (Table 1). This is also consistent with our in-house intraoperative procedure, in which lateral screw deviations are not targeted but tolerated with sufficient clinical stability, as well as medial screw deviations without neurological limitations.

The placement of pedicle screws during dorsal instrumentation is challenging, especially because intraoperative visualization of the pedicle screw position using conventional fluoroscopy is considered to be only of limited reliability [10]. Corresponding malpositions of pedicle screws in all directions (medial, lateral, cranial and caudal) have been described, but medial and lateral deviations can be clinically relevant. While medial pedicle screw deviations may be associated with injuries of important nervous structures of the spinal cord [3], lateral pedicle screw dislocations are considered to be negative for the biomechanical stability of the dorsal instrumentation. Due to these described factors, a pedicle screw position centrally in the pedicle is desired by the surgeon but is not easy to achieve.

Malposition rates (1.7–31% and 15–72%, respectively) in postoperative CT with the free-hand technique without intraoperative imaging or only with the use of conventional fluoroscopy can be significant [10, 18]. Accordingly, there is an increasing desire for improved intraoperative imaging. Therefore, CT has been established as the standard method in the assessment of pedicle screw position [5–8], but the intraoperative availability is low. Therefore, other techniques, such as CBCT, have been developed to close this intraoperative gap. Additionally, good (type A + B according to Gertzbein) pedicle screw positions have been observed in 88.8–94.1% patients after dorsal instrumentation with intraoperative imaging by a CBCT scan [15, 19]. Moreover, it has been shown that intraoperative imaging with CBCT is not inferior to intraoperative CT [20, 21]. However, a disadvantage of a CBCT scan compared to conventional fluoroscopy is the higher radiation exposure [22], which is certainly not negligible.

Studies that specifically examine the intraoperative value of CBCT for dorsal instrumentation in patients with obesity (BMI > 30 kg/m^2^) are not available in the current literature. Independent of intraoperative imaging, there are few studies examining the pedicle screw position during dorsal instrumentation in patients with obesity. Winder et al. observed a nonsignificantly higher rate of pedicle screw perforation in patients with obesity after dorsal instrumentation and intraoperative conventional fluoroscopy [14]. Additionally, Park et al. investigated the pedicle screw position after dorsal instrumentation with conventional fluoroscopy in patients with obesity with a BMI > 30 kg/m^2^ who received 89 pedicle screws; the authors did not observe a significant difference in postoperative pedicle screw positions compared to normal-weight patients [13].

However, a significantly higher inaccuracy in pedicle screw positioning could be observed in patients with obesity using intraoperative CBCT imaging in contrast to normal-weight patients. Simultaneously, this finding could not be monitored in pedicle screw positioning in normal-weight and patients and with obesity when using conventional fluoroscopy. Nevertheless, the results suggest that regardless of body weight, the accuracy of the pedicle screw positions was reduced when using only conventional fluoroscopy in comparison with an additional usage of intraoperative CBCT imaging. However, since precise pedicle screw placement goes along with an improved stability of the dorsal instrumentation, an optimal pedicle screw positioning appears relevant in obese patients, who particularly seem to benefit from intraoperative CBCT imaging.

But, the results of this study must be interpreted in light of several limitations. (1) This is a retrospective study with a small sample size. Accordingly, it is difficult to draw a definitive conclusion regarding the influence of BMI on postoperative pedicle screw position. Furthermore, due to the small subgroup sizes among the patients with obesity, no significant difference can be observed between these groups. (2) The distribution of pedicle screws showed that more pedicle screws were inserted in the cervical spine among the normal-weight patients than among patients with obesity. Considering that the screw–pedicle ratio in the cervical spine is less favorable than in the thoracic and lumbar spine, this may have led to an increased screw perforation postoperatively in the cohort of normal-weight patients. (3) Despite intraoperative CBCT imaging, postoperative screw perforations were also observed. These were also registered intraoperatively. However, as these mainly showed lateral deviation and did not lead to neurological or vascular complaints, they were tolerated. (4) Only the radiologic results of intraoperative 3D imaging were assessed but not the functional outcomes. All screws that would have caused neurologic or vascular complications would have been revised. Since no clinically relevant complications were observed, no screw was revised based on postoperative CT imaging. (5) Two different types of CBCT were used in this study: Cios Spin (Siemens, Erlangen, Germany) and ARCADIS Orbic 3D (Siemens, Erlangen, Germany). The quality of imaging of the Cios Spin was superior to that of the ARCADIS Orbic 3D. Even if not specified, a certain influence cannot be excluded with absolute certainty.

## Conclusion

CBCT imaging in dorsal instrumentation can lead to an improved pedicle screw position independent of body weight in patients with obesity and normal-weight patients. However, patients with obesity showed significantly worse pedicle screw positions postoperatively after dorsal instrumentation with intraoperative CBCT imaging than normal-weight patients.

## Data Availability

The datasets used and analyzed during the current study are available from the corresponding author on reasonable request.
